# On the Use of Stramonium in Laryngismus Stridulus

**Published:** 1844-07

**Authors:** J. P. Epperson

**Affiliations:** Pulaski, Tennessee


ART. II.—*On the use of Stramonium in Laryngismus Stridulus.* By Dr. J. P. EPPERSON, of Pulaski, Tenn.

The father of the children whose cases are the subject of this communication, is a stout, healthy negro man, about 35 years old. The mother is a large and likely mulatto woman, about 28 years of age. From the age of eight years, till puberty she was affected with violent and obstinate spells of sciatica, and was treated at the latter age, by large doses of stramonium, I judge from the described effects of the medicine used, and from some knowledge of the practice of the physician who attended her; but this I cannot certainly ascertain because the physician referred to died several years ago. Whether this woman's disease or the treatment of it had any influence on her children, cannot be known, but I state the fact, because I have had a vague conjecture that the treatment referred to may have had some effect on the offspring.

This woman has had nine children. The first child was healthy and remains so. The last is healthy yet, and is now several months old. But all the seven children between the first and last, were subject to spasmodic croup. They all appeared well for sometime after birth, until they were attacked with the disease, which occurred about the seventh week in two, and from the sixth to the eighth month of their age in the other five.

They were usually attacked suddenly, with great difficulty of breathing, amounting to impending suffocation, attended with a peculiar loud crowing sound, resembling that heard in pertussis. The paroxysms were usually excited by fright, an attempt to cry, an effort to swallow, or waking from sleep, and lasted from a few seconds to a few minutes only, and returned frequently during the twenty-four hours. But in the intervals the patients were well and lively. When the disease had continued thus for a short time, symptoms of general spasms appeared, such as strabismus, convulsive contractions of the fingers and toes, particularly contraction ot the thumbs upon the palms of the hands, and of the great toes towards the bottoms of the feet. These symptoms though continuous were increased by the paroxysms. Occasionally there were slight spasms of the legs and arms.

The first three of the seven cases terminated fatally in the 12th or 15th month after birth, before I became acquainted with the family. They were treated, I understand, by laxatives, alterants, and warm baths. I prescribed for the other four cases, and they all recovered in their second year, and are now healthy children. My mode of treatment was this: in addition to the treatment mentioned above, I gave from a quarter of a grain to a grain, according to age and other circumstances, of the extract of stramonium, three times a day until its peculiar effects on the system were decidedly manifested, at which time the paroxysms of the disease ceased in every case, and did not return for several days afterwards. Whenever they did recur the medicine was resumed, and continued as before, until they again disappeared. This alternation of the exhibition, and the discontinuance of the remedy was pursued through the whole course of every case.

I found that the disease gradually gave way under it, but that the *fits* were induced by any strongly exciting cause during the whole period of dentition. At one time, having none of the stramonium, I gave, instead, about the same doses of the extract of belladonna, but with no apparent benefit.

These circumstances attending the cases make the beneficial effects of stramonium very evident:

1st. The seven cases were in successive children of the same parents, and therefore more likely to have been similar in all respects than if they had belonged to different families.

2d. The striking fact that in the first three cases, without the remedy all died, and that in the next four, with the remedy all recovered.

3d. The obvious fact noticed at different times in each successful case, that whenever the specific effects of the medicine were manifested the paroxysms ceased, and did not return for sometime after it was discontinued. This was so obvious that the attendant quit all auxiliary means of treatment, confident that she could control the disease by stramonium alone.

*Pathology.*—The subjects of the three fatal cases were not examined *post-mortem;* and if they had been examined it is doubtful whether any light would have been thrown on the nature of the disease.

In the four cases which I saw the same symptoms were present which induced Cheyne and others to refer them to lesion of the brain. But there were no visibly enlarged glands, or other mechanical obstructions of the air-tubes; causes to which some physicians have ascribed the symptoms of the disease. I think the best that can be said in relation to the pathology of the malady, outside of extensive post-mortem observations, is, that it consists in a peculiar irritation of the nervous system, originating either in the brain, or in the nerves themselves, and manifested more particularly in the glottis, becase that part is endowed with more sensibility.

And, owing to the smallness and importance of that aperture, some degree of irritation which might be innocent in other parts of the system, is fatal in it.

Pulaski, May, 29, 1844.

